# Age modification of diabetes-related hospitalization among First Nations adults in Alberta, Canada

**DOI:** 10.1186/1758-5996-6-108

**Published:** 2014-10-02

**Authors:** David JT Campbell, Sarah L Lacny, Robert G Weaver, Braden J Manns, Marcello Tonelli, Cheryl Barnabe, Brenda R Hemmelgarn

**Affiliations:** Department of Community Health Sciences, University of Calgary, Calgary, AB Canada; Department of Medicine, University of Calgary, Calgary, AB Canada; Interdisciplinary Chronic Disease Collaboration, Calgary, AB Canada; Institute of Public Health, University of Calgary, Calgary, AB Canada; Libin Cardiovascular Institute, University of Calgary, Calgary, AB Canada; Department of Medicine, University of Alberta, Edmonton, AB Canada; Foothills Medical Centre, Room C210, 3330 Hospital Dr. NW, Calgary, AB T2N 1N4 Canada

**Keywords:** American indian, First Nations, Hospitalization, Diabetes mellitus, Risk adjustment

## Abstract

**Background:**

We sought to determine the modifying effects of age and multimorbidity on the association between First Nations status and hospitalizations for diabetes-specific ambulatory care sensitive conditions (ACSC).

**Findings:**

We identified 183,654 adults with diabetes from Alberta Canada, and followed them for one year for the outcome of hospitalization or emergency department (ED) visit for a diabetes-specific ACSC. We used logistic regression to determine the association between First Nations status and the outcome, assessing for effect modification by age and multimorbidity with interaction terms. In a model adjusting for age, age^2^, baseline A1c, duration of diabetes, and multimorbidity, First Nations people were at greater risk than non-First Nations to experience a diabetes-specific hospitalization or ED visit (unadjusted odds ratio [OR] 3.74; 95% confidence interval [CI]: 3.45-4.07). After adjustment for relevant covariates, this association varied by age (interaction: p = 0.018): adjusted OR 3.94 (95% CI: 3.11-4.99) and 5.74 (95% CI: 3.36-9.80) for First Nations compared to non-First Nations at ages 30 and 80 years, respectively.

**Conclusions:**

Compared with non-First Nations, older First Nations patients with diabetes are at greater risk for diabetes-specific hospitalizations. Older First Nations patients with diabetes should be given priority access to primary care services as they are at greatest risk for requiring hospitalization for stabilization of their condition.

## Background

The Canadian Aboriginal population is a diverse one. The Canadian constitution recognizes three distinct populations of Aboriginal peoples: First Nations, Inuit and Métis [1]. 65% of the Canadian Aboriginal population identify as First Nations [2]. Compared to non-First Nations, First Nations people in North America have more than a two-fold increased prevalence of diabetes [3], and experience more diabetes-specific complications requiring hospitalization [4, 5]. Alberta’s First Nations population is heterogeneous and comprises 44 distinct First Nations with a total of over 100,000 individuals registered under the Federal Indian Act. Nearly 60% of First Nations people live on one of 134 designated reserves while the remainder live off-reserve [6].

It is not well known whether certain subsets of the First Nations population with diabetes are at greater risk of hospitalization; though among the non-First Nations population risk has been noted to decrease with advancing age and increases with multiple comorbid conditions [7]. Given the increasing life expectancy and incidence of chronic conditions among First Nations, the potential modifying effect of age and multimorbidity is particularly relevant [8].

Using a population-based cohort we sought to determine the association between First Nations status and risk of hospitalization for diabetes-specific ambulatory care sensitive conditions (ACSCs), and to assess whether this risk varies with age and multimorbidity.

## Methods

We used administrative data from Alberta Health and the Alberta Kidney Disease Network for our population-based study [9]. For the period from Apr. 1, 1994 to Apr. 1, 2009 we obtained data on hospital admissions, physician visits, and emergency department visits from Alberta Health administrative data files. We also obtained laboratory data from a province-wide repository that captures data for all Albertans who undergo inpatient or outpatient laboratory testing. This data was de-identified prior to the researchers being granted access. A validated algorithm using ICD-9 codes from the administrative database was used to define a cohort of adults (≥18 years) with a diagnosis of diabetes between April 1, 1994 and March 31, 2008 [10]. An individual was classified as having diabetes if they were found to have either one hospital discharge abstract with a diagnosis of diabetes or a diabetes-related code on two physician claims within 2 years. This algorithm does not distinguish between individuals with type 1 and type 2 diabetes and largely eliminates cases of gestational diabetes.

### Exposure variable

In our dataset, First Nations status reflected registration under the Federal Indian Act. In the 2006 Canadian Census, 81% of those who self-identified as First Nations were registered with the Federal Indian Act [11].

### Outcome variable

The outcome was hospitalization or emergency department (ED) visit for a diabetes-specific ACSC during a one-year period (April 1, 2008 to March 31, 2009), and included hypoglycemic events, diabetic ketoacidosis or hyperglycemic hyperosmolar non-ketotic states [12]. ACSCs represent conditions which, if managed optimally in the outpatient setting, should not result in hospitalization or ED visit [13, 14]. ACSCs are commonly used as a marker of quality care for patients with chronic disease [4], and diabetes specifically [9, 13, 15].

### Covariates and other variables of interest

Demographic data, including age and sex, were determined from the Alberta Health Registry File. The presence of comorbid conditions was generated using validated ICD-9-CM and ICD-10 coding algorithms [16]. Specifically, we used the Charlson Comorbidity Index to identify comorbidities [17]. Patients with a Charlson score greater than 2, in addition to diabetes, were classified as having multimorbidity. Hypertension was identified as a separate comorbidity. Baseline glycated hemoglobin (A1c) level was obtained from a provincial laboratory repository using the most recent A1c assessment prior to April 1, 2008 [18]. Duration of diabetes was the length of time between the diabetes diagnosis date and the index date of April 1, 2008.

#### Statistical analysis

We used logistic regression to determine the odds ratios (ORs) of a hospitalization or ED visit for a diabetes-specific ACSC for First Nations compared with non-First Nations, adjusting for demographic and clinical characteristics. We used interaction terms to assess for effect modification by age (age*First Nations status) and by multimorbidity status (multimorbidity*First Nations status), as well as joint modification by both age and multimorbidity (age*multimorbidity*First Nations status). Based on the literature [14, 19, 20], we considered the following potential confounders: duration of diabetes, baseline A1c, hypertension, sex, multimorbidity status, age, and a quadratic age term (age*age). Age, age^2^ and A1c were treated as continuous variables in analyses. We used a backwards stepwise elimination technique to obtain the most parsimonious model that was still hierarchically well formulated. Ethics approval was obtained from the University of Calgary Research Ethics Board.

## Results

Compared to non-First Nations, First Nations adults with diabetes were more likely to: be younger, be female, have multiple medical conditions, have higher baseline A1c, and have a longer duration of diabetes (Table 1).

**Table 1:** **Baseline characteristics**

	First nations	Non-first nations	p-value
	N = 8007	N = 175,647	
Persons having a hospitalization for diabetes-related ACSC N (%)	710 (8.9%)	4450 (2.5%)	<0.001†
Age, years Mean (SD)	53.4 (13.9)	61.6 (15.1)	<0.001*
Gender, women N (%)	4585 (57.3)	82,573 (47.0)	<0.001†
Multimorbidity			
Charlson Comorbidity Indexǂ			
0–1, N (%)	5367 (67.0)	120,390 (68.5)	0.004†
2+, N (%)	2640 (33.0)	55,257 (31.5)	
Hypertension N (%)	4353 (54.4)	115,047 (65.5)	<0.001†
Baseline A1c, % Mean (SD)	7.85 (2.14)	7.24 (1.56)	<0.001*
Duration of diabetes, years	7.18 (4.44)	6.63 (4.39)	<0.001*
Mean (SD)			

*Comparison by *t*-test.

† Comparison by chi-square test.

ǂ comorbidities considered included: myocardial infarct, congestive heart failure, peripheral vascular disease, dementia, cerebrovascular disease, chronic lung disease, connective tissue disease, peptic ulcer disease, chronic liver disease, malignancies, and chronic kidney disease.

**Figure 1:**
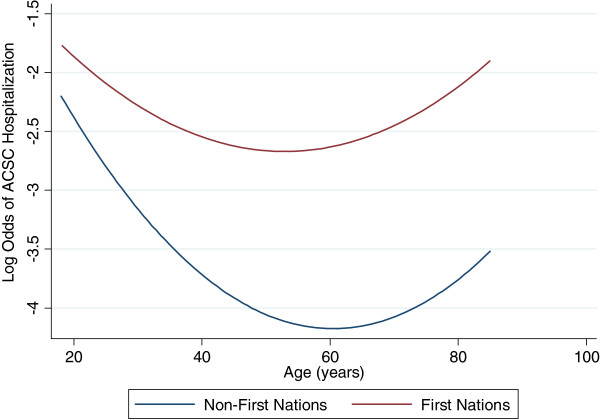
**Log odds of diabetes-specific ACSC hospitalization.**

**Figure 2:**
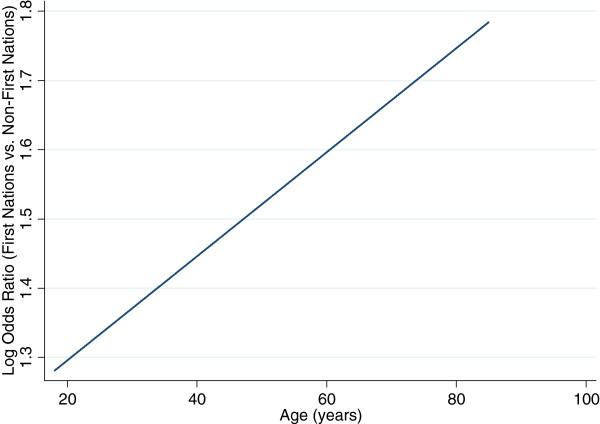
**Log odds ratio of diabetes-specific ACSC hospitalization for First Nations individuals compared to non-First Nations individuals.**

First Nations adults had almost four times the odds of having a hospitalization or ED visit for a diabetes-specific ACSC compared with non-First Nations (unadjusted OR: 3.74; 95% CI: 3.45-4.07). Because of the necessity to adjust for age^2^, the adjusted odds of hospitalization for both groups follows a parabolic path (Figure 1). The relationship is such that, among adults with diabetes, regardless of First Nations status, the odds of hospitalization decreases with age until 60 years and then increases with advancing age. At all ages, non-First Nations adults with diabetes have significantly higher log odds of hospitalization for diabetes-related ACSC.The nature of the relationship between First Nations status and hospitalization or ED visit was significantly modified by age (age*First Nations status p = 0.018), with increased excess risk at older ages. This is represented by the vertical distance between the two lines in Figure 1 – these lines are much closer together at younger ages than at older ages. The relationship between age and log odds of diabetes-related ACSC hospitalization is a linear one (Figure 2).

The adjusted OR of hospitalization increases concomitantly with age (Table 2). The adjusted ORs (95% CI) for First Nations compared with non-First Nations varies linearly from 3.94 (3.11-4.99) at age 30 to 5.74 (3.36-9.80) at age 80 years.

**Table 2:** **Adjusted age-specific odds ratio of diabetes-specific ACSC hospitalization**

Odds ratio (95% CI)	Age (years)		
	30	55	80
First Nations Status, including age interaction	3.94 (3.11-4.99)	4.75 (3.24-6.97)	5.74 (3.36-9.80)
Age	0.80 (0.75-0.85)	0.67 (0.60-0.75)	0.56 (0.48-0.65)
Baseline A1c	1.33 (1.31-1.35) per % increase in A1c		
Duration of diabetes	1.11 (1.10-1.11) per year of exposure		
Multimorbidity	2.05 (1.92-2.18)		

Multimorbidity did not modify the relationship between First Nations status and hospitalization or ED visit for a diabetes-specific ACSC (interaction: p = 0.197).

## Discussion

In this population-based cohort of patients with diabetes the risk of hospitalization or ED visit for a diabetes-specific ACSC was consistently higher for First Nations compared with non-First Nations – and the magnitude of the excess risk increased with age. By age 80 the risk was almost six times higher for First Nations compared to non-First Nations, even after adjusting for multimorbidity and other factors. Given the aging First Nations population and the increasing prevalence of diabetes, these results are particularly relevant and worthy of further exploration.

Our study supports previous findings suggesting that First Nations are at higher risk of hospitalizations for diabetes-specific ACSCs than non-First Nations [4, 15, 21]. However, this is the first study, to our knowledge, to further assess this risk by age and multimorbidity status. These findings have important implications for provision of diabetes care. Providers should be aware that among First Nations patients, those with advanced age may have a higher excess risk and thus warrant closer follow-up.

A strong association between diabetes incidence and age is known to exist, where rates of diabetes increase concordantly with age [22, 23]. However, the risk of diabetes-specific ACSC hospitalizations among the non-First Nations population with diabetes has been shown to decrease with age and multimorbidity [7]. In contrast, by adjusting for age in a non-linear fashion (age^2^), we were able to identify that the risk of hospitalization decreases until age 60 and thereafter increases with advancing age. The reason for this pattern may be improved disease management through adulthood which is later complicated by incident comorbid conditions with advancing age.

Furthermore, we found that the excess risk of diabetes-specific ACSC hospitalization among First Nations adults compared to non-First Nations adults increased with advancing age, and did not vary by the presence of multimorbidity. The reasons for these excess risks in First Nations patients, as compared to non-First Nations patients are complex and have their roots in social and historical inequities [24]. These inequities may lead to the accumulation of a variety of factors over time, which may explain the excess risk in older First Nations adults. These factors include: poor glycemic control, inconsistent medication adherence, inadequate specialized care for diabetes-specific problems, and, consequently, an increased risk of developing long-term complications and adverse health outcomes [4, 25]. While life expectancy in general is increasing, the First Nations population in particular has experienced an increase in life expectancy [8]. As they age this population is at a particularly high risk of adverse outcomes related to their diabetes in the future. We feel that the results of our study are likely to apply to other Canadian First Nations populations outside of Alberta.

A strength of our study was the population-based nature of our data, which allowed us to define a large and representative cohort of patients with diabetes. However, there are also inherent limitations based on the data sources, including potential misclassification of outcomes or comorbidities. However, such misclassification is unlikely to have differed by First Nations status, and therefore (if present) would bias the results towards the null. We did not have data on measures of socioeconomic status (income, social environment, education, etc.…), therefore we could not fully adjust our models for potential confounding variables which may partially contribute to differences seen in our analysis. We were also unable to assess whether the risk of hospitalization varied by diabetes type (Type 1 vs. Type 2), this would be problematic if type 2 diabetes were known to be associated with higher rates of hospitalizations as First Nations patients are much more likely to have type 2 diabetes, compared with the general population [26] and this may result in differential misclassification. However, the reason for hospitalization and length of stay is known to vary by type of diabetes, but the number of hospitalizations is not particularly variable, and in fact, those with Type 1 diabetes seem to be at higher risk for hospitalization [27]. Therefore, the inability to distinguish between Type 1 and Type 2 diabetes is unlikely to bias the results of our study.

## Conclusion

Our study highlights a substantial difference in risk of hospitalization or ED visit for diabetes-specific ACSC by age for First Nations people, as compared to non-First Nations people. Given the aging of the First Nations population, this risk is particularly concerning and warrants further study.
